# Increased *N*^*6*^-methyladenosine in Human Sperm RNA as a Risk Factor for Asthenozoospermia

**DOI:** 10.1038/srep24345

**Published:** 2016-04-13

**Authors:** Ying Yang, Wei Huang, Jing-Tao Huang, Fan Shen, Jun Xiong, Er-Feng Yuan, Shan-shan Qin, Ming Zhang, Yu-Qi Feng, Bi-Feng Yuan, Song-Mei Liu

**Affiliations:** 1 Center for Gene Diagnosis, Zhongnan Hospital of Wuhan University, Donghu Road 169#, Wuhan, 430071, P.R. China; 2 Key Laboratory of Analytical Chemistry for Biology and Medicine (Ministry of Education), Department of Chemistry, Wuhan University, Wuhan, 430072, P. R. China; 3 Reproductive Medicine Center, Zhongnan Hospital of Wuhan University, Donghu Road 169#, Wuhan, 430071, P.R. China

## Abstract

Male infertility is a worldwide medical problem. Asthenozoospermia is a common cause of infertility. Epigenetic modifications of DNA and histones have been shown to influence human infertility, but no research has explored whether *N*^6^-methyladenosine (m^6^A) level in RNA is associated with asthenozoospermia. Here, we collected a total of 52 semen samples, including 20 asthenozoospermia patients and 32 healthy controls. An LC-ESI-MS/MS method was used to detect m^6^A contents in sperm RNA, and real-time PCR was performed to determine the mRNA expression of demethylase (FTO, ALKBH5), methyltransferase (METTL3, METTL14, WTAP) and an m^6^A-selective-binding protein (YTHDF2). We found that m^6^A content (*p = *0.033) and the mRNA expression of *METTL3* (*p = *0.016) and *METTL14* (*p* *=* 0.025) in asthenozoospermia patients were significantly higher than those of controls. Increased m^6^A content was a risk factor for asthenozoospermia (odds ratio (OR) 3.229, 95% confidence interval (CI) 1.178 – 8.853, *p* = 0.023). Moreover, m^6^A content was correlated with the expression of *METTL3* (r = 0.303, *p* = 0.032) and with sperm motility (progressive motility: r = −0.288, *p* = 0.038; non-progressive motility: r = −0.293, *p* = 0.037; immotility: r = 0.387, *p* = 0.005). Our data suggest that increased m^6^A content is a risk factor for asthenozoospermia and affects sperm motility. Methyltransferases, particularly METTL3, play key roles in increasing m^6^A contents in sperm RNA.

Human infertility is a prevalent medical problem, which affects approximately 15% of couples worldwide^1^,^2^. Tragically, the infertility rates in some developing countries can reach 30%^3^. Male infertility contributes to more than 50% of all cases of infertility^4^. Optimal male fertility depends on an adequate number of normal, motile, mature and physiologically functional sperm^5^, which may be influenced by many factors, such as genetic disorders, infections of the genital tract, medical intervention and environmental degradation^1^. Decreased semen quality and defective sperm function appear to be common causes of male infertility^1^. Many current studies have probed the pathogenesis of infertility, but the interference of different epigenetic alterations in infertility is not fully understood.

Epigenetics is defined as a heritable change affecting gene expression that is not caused by DNA sequence alterations^6^. Epigenetic modifications of DNA and histones have been confirmed as having significant roles throughout spermatogenesis^2^. There are two characteristic features of sperm epigenetic programming. The first feature is a major removal of epigenetic markers occurring in the primordial germ cells (PGCs). The second feature is the reorganization and condensation of the germ cell genome during postmeiotic maturation, including DNA methylation and histone modifications during spermiogenesis^2^. Abnormal sperm DNA methylation levels are related to altered semen parameters^7^. Moreover, many specific RNAs, mRNAs, miRNAs, and Piwi-interacting RNAs (piRNAs) in sperm are vital for male fertilization^8^,^9^,^10^,^11^. In addition to DNA and histone modifications, epigenetic modifications of RNA have been proposed to be another layer of epigenetic regulation. Up to date, more than 100 types of RNA modifications, occurring in mRNA, tRNA, rRNA and small nuclear RNA (snRNA), have been identified^12^. Among these modifications, *N*^6^-methyladenosine (m^6^A) modification is the most prevalent in mammalian mRNA^13^. Despite m^6^A modification being first reported in early 1970s^14^, its biological function and significance in sperm RNA are largely unknown.

*In vivo*, the dynamic regulation of m^6^A modification plays important roles through the functional interplay among m^6^A methyltransferases and demethylases^15^. The methyltransferase complex, which is composed of methyltransferase like 3 (METTL3), methyltransferase like 14 (METTL14) and Wilms tumor 1 associated protein (WTAP), has been identified as a crucial factor catalyzing the formation of m^6^A with S-adenosyl-L-methionine (SAM) as the methyl donor^16^. In contrast to methyltransferases, two demethylases, the alpha-ketoglutarate- and Fe^2+^ -dependent dioxygenase fat mass and obesity associated protein (FTO) and AlkB family member 5 protein (ALKBH5), have similar functions as the TET enzyme in DNA demethylation^17^; i.e., both FTO and ALKBH5 can remove the methyl group from m^6^A in RNA^18^,^19^. In this dynamic modification, methyltransferases act as ‘writers’, demethylases serve as ‘erasers’, and m^6^A-selective-binding proteins(YTHDF) represent ‘readers’ of m^6^A in mRNA. More recently, the YTHDF domain family 2 protein (YTHDF2) has been shown to regulate RNA stability, translation, splicing, transport and localization through selective recognition of methylated RNA^20^,^21^.

Unlike DNA methylation, very few studies directly focused on the role of RNA methylation in human disease, particularly m^6^A. Recent advances have demonstrated that *FTO* expression and m^6^A levels are inversely correlated during adipogenesis^22^ and the *FTO* gene encoding an m^6^A demethylase is also associated with human obesity^23^. We have reported that the increased mRNA expression of *FTO* contributed to the reduction of m^6^A in type 2 diabetes mellitus^24^. However, the association of m^6^A with human fertility can be traced back to 1997, when Bokar and colleagues first revealed using Northern blot analysis that the mRNA of *METTL3* (*MT-A70*) was expressed in a wide variety of human tissues, with the highest levels being in testis^25^. In addition, a recent animal study documented that *Alkbh5*-deficient male mice showed increased m^6^A in mRNA and impaired fertility due to compromised spermatogenesis^19^. Based on these experimental results, we hypothesize that m^6^A modification is associated with human asthenozoospermia and plays a role in male infertility.

To verify our hypothesis, in the current study, we first detected the m^6^A contents in sperm RNA from asthenozoospermia patients and healthy controls. Compared with the controls, m^6^A content in asthenozoospermia patients was significantly increased, which is a risk factor for asthenozoospermia. To better understand this phenomenon, we performed real-time PCR to examine the mRNA expression of key genes involved in m^6^A regulation. Our results indicated that the mRNA expression of *METTL3* and *METTL14* was higher than that of controls. In addition, m^6^A content was closely related to the mRNA expression of *METTL3* and clinical parameters of sperm.

## Results

### Clinical parameters of semen

The clinical parameters of semen from 20 asthenozoospermia patients and 32 healthy controls are given in Table 1. Student’s t test indicated that there was no significant difference in semen pH and teratozoospermia index (TZI) between the two groups; nevertheless, asthenozoospermia patients showed significantly lower sperm concentration (39.2 × 10^6^/mL vs. 96.6 × 10^6^/mL), progressive motility (8.6% vs. 24.7%), non-progressive motility (19.8% vs. 44.5%) and normal morphology (5.1% vs. 8.2%) and higher sperm immotility (72.4% vs.30.8%) and sperm deformity index (1.1% vs. 1.0%) compared with the controls.

### LC-ESI-MS/MS method development

The linearity of this method was investigated using 40 pmol rA standard supplemented with m^6^A at different amounts ranging from 8 fmol to 800 fmol (Table S1). The calibration curves were constructed by plotting the mean peak area ratio of m^6^A/rA versus the mean molar ratio of m^6^A/rA based on data obtained from triplicate measurements. The results showed linearity within the range of 0.02–2% (molar ratio of m^6^A/rA) with the coefficient of determination (R^2^) higher than 0.9977 (Table S1). Limits of detection (LOD) and limits of quantification (LOQ) for m^6^A were calculated as the amounts of the analytes at signal/noise ratios of 3 and 10, respectively. The LOD and LOQ were 1.2 fmol and 4.0 fmol for m^6^A, respectively (Table S1). Validation of the method was accomplished using the synthesized m^6^A-containing oligonucleotide by comparing the measured m^6^A content to the theoretical m^6^A content (Table S2). m^6^A was determined from RNA hydrolysis products with RSDs being 1.8–10.6% and REs being 2.7–12.1% (Table S3), indicating high accuracy and precision for the determination of m6A by LC-ESI-MS/MS.

### m^6^A contents increased in asthenozoospermia

**Figure 1:**
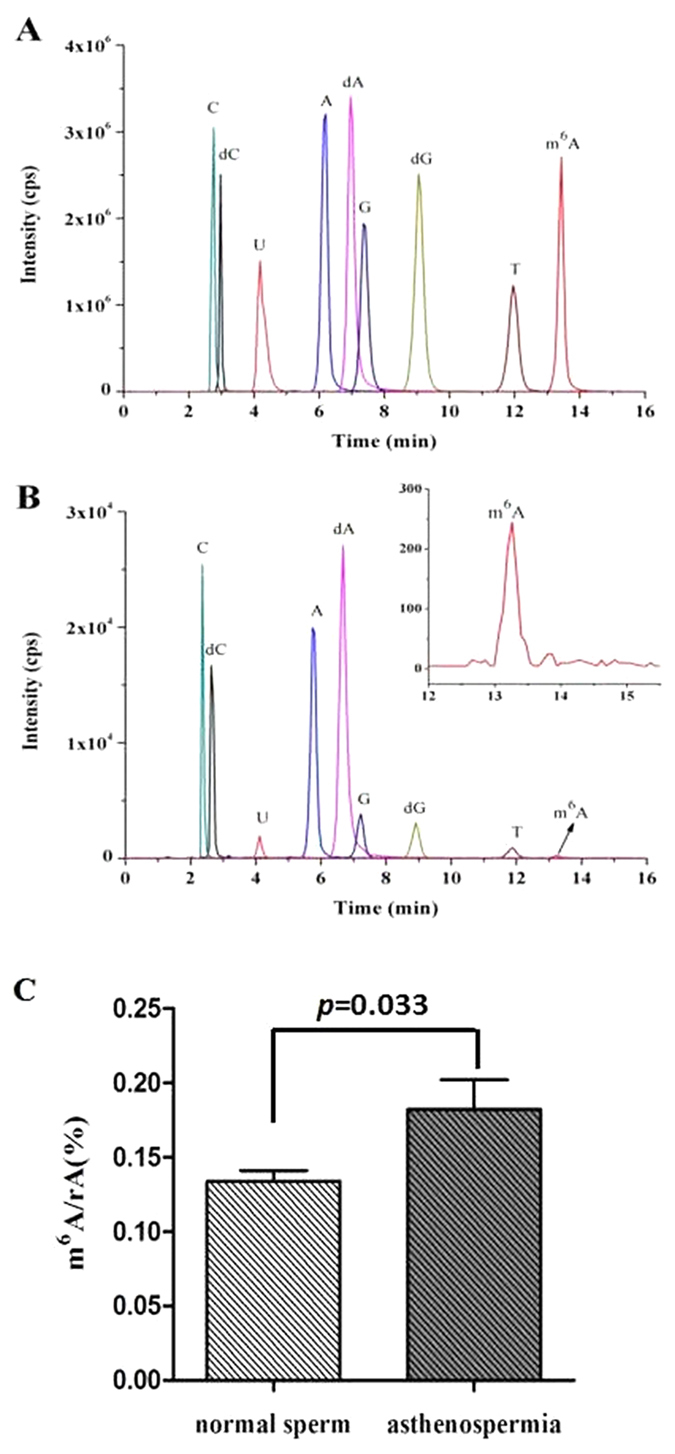
The MRM chromatograms of nucleosides and difference of m^6^A contents between the two groups. (**A**) Standard nucleosides. (**B**) 50 ng sperm RNA from an asthenozoospermia patient. Shown in inset is the enlargement chromatogram of m^6^A. (**C**) Comparative analysis of m^6^A contents in sperm RNA from asthenozoospermia patients (n = 20) and controls (n = 32).

To investigate whether m^6^A modification differs in asthenozoospermia patients and controls, the m^6^A contents of 52 sperm RNA samples, including 20 asthenozoospermia patients and 32 controls, were analyzed by LC-ESI-MS/MS in multiple reaction monitoring (MRM) mode (Table S4). The MRM chromatograms of 9 standard nucleosides and the hydrolysis products of 50 ng sperm RNA from an asthenozoospermia patient are shown in Fig. 1A,B. The average contents of m^6^A in asthenozoospermia patients and healthy controls were 0.18 ± 0.02% and 0.13 ± 0.01%, respectively. Student’s t test indicated asthenozoospermia patients had higher m^6^A contents (*p* = 0.033), as shown in Fig. 1C. Importantly, logistic regression analysis revealed that increased m^6^A content in sperm RNA was a risk factor for asthenozoospermia (odds ratio (OR) 3.229, 95% confidence interval (CI) 1.178 – 8.853, *p* = 0.023).

### mRNA expression of the regulatory genes of dynamic RNA methylation and its correlation with m^6^A contents

**Figure 2:**
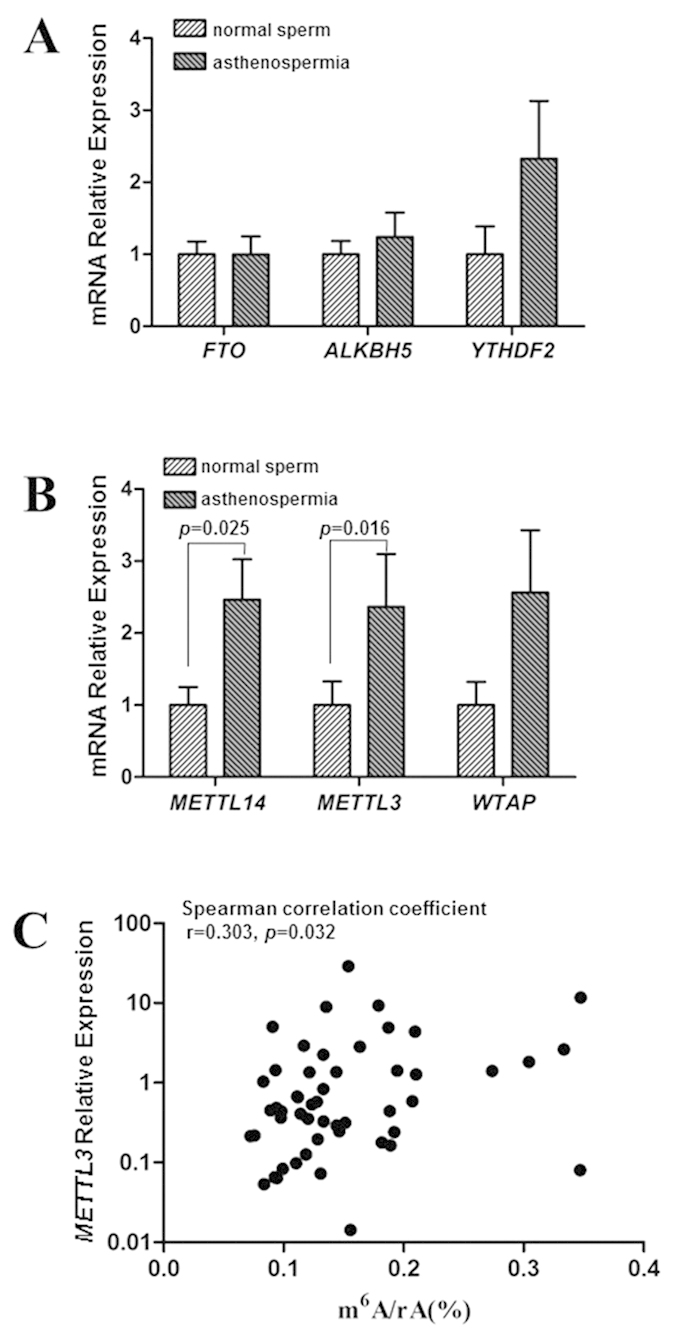
mRNA expression of the regulatory genes of dynamic RNA methylation and the correlation of *METTL3* expression with m^6^A contents. (**A,B**) Gene expression levels of the regulatory genes of dynamic RNA methylation, normalized to GAPDH, were examined by real-time PCR. Data are expressed as means ± SEM. (**C**) Spearman correlation analysis between m^6^A contents and mRNA expression of *METTL3* in all subjects.

To address why m^6^A contents increased in asthenozoospermia patients, we utilized real-time PCR to determine the mRNA expression levels of the core regulatory genes that would be engaged in dynamic m^6^A modification of RNA (Fig. S1). We used Student’s t test to compare the differences in *ALKBH5* and *METTL14* between the two groups, and the Mann-Whitney U test for *FTO, METTL3, WTAP and YTHDF2* comparisons. Asthenozoospermia patients showed significantly higher mRNA expression levels of the methyltransferases, including *METTL3* (*p* = 0.016) and *METTL14* (*p* = 0.025) compared to the healthy controls (Fig. 2B). The average mRNA expression levels of *METTL3 and METTL14* were 2.4-fold (*p* = 0.016) and 2.5-fold (*p* = 0.025) higher, respectively, than those of controls (Fig. 2B). However, there was no significant difference in *FTO, ALKBH5*, *WTAP* or *YTHDF2* mRNA levels between asthenozoospermia patients and controls (Fig. 2A,B). Interestingly, Spearman correlation analysis indicated that the mRNA expression of *METTL3* (r = 0.303, *p* = 0.032) (Fig. 2C), *WTAP* (r = 0.324, *p* = 0.023) and *YTHDF2* (r = 0.338, *p* = 0.014) (Fig. S2) associated with the contents of m^6^A in sperm RNA; while no correlation was found between *FTO* expression and m^6^A contents (r = 0.268, *p* = 0.057); Pearson correlation analysis revealed neither *ALKBH5* (r = 0.055, p = 0.700) nor *METTL14* (r = 0.194, *p* = 0.172) was correlated with the contents of m^6^A (Fig. S2). Our data suggested that the increased m^6^A contents in asthenozoospermia patients might be caused by the upregulation of methyltransferase encoding genes, particularly *METTL3*.

### m^6^A content associated with clinical parameters of sperm

To test whether m^6^A content is related to the clinical parameters of sperm, we performed Pearson correlation analysis to evaluate the correlation between m^6^A content and several clinical parameters. m^6^A content was negatively correlated with sperm progressive motility (r = −0.288, *p* = 0.038) and non-progressive motility (r = −0.293, *p* = 0.037), and positively correlated with sperm immotility (r = 0.387, *p* = 0.005) (Table 2). No correlation was found between m^6^A contents and other clinical parameters.

## Discussion

The majority of male infertility results from asthenozoospermia. Despite tremendous efforts to characterize the genetic basis for this disorder, almost half of male infertility cases are of unknown etiology^26^. So far, few reports described RNA epigenetic alterations in human asthenozoospermia. Given the importance of m^6^A in RNA stability, splicing regulation, miRNA attenuation, RNA editing prevention, disease acceleration and epigenome control^27^, we first established a highly sensitive LC-ESI-MS/MS method to measure the m^6^A content in sperm RNA.

Even though there are many methods to detect m^6^A in RNA, such as MeRIP-Seq (m^6^A-specific methylated RNA immunoprecipitation combined with next-generation sequencing)^28^, the facile enzymatic ligation-based method^29^, the selective Polymerase method^30^, SMRT reverse transcription^31^ and the LC-ESI-MS/MS method^24^. We chose the LC-ESI-MS/MS method to determine m^6^A contents in the semen samples as its high sensitivity, relative low cost and satisfying qualitative and quantitative ability.

One of the most obvious and distinctive features of asthenozoospermia is weak sperm motility, which is defined as the proportion of progressively motile sperm. In our study, correlations existed between m^6^A contents and sperm progressive motility, non-progressive motility and immotility, suggesting that increased m^6^A in sperm RNA from asthenozoospermia patients might be a leading cause of the reduction of sperm motility. Previous studies have demonstrated that the nucleus of mature sperm contains a complex population of RNAs^8^, and differential expression of sperm mRNA is correlated with sperm motility and male reproduction^32^. We also found that m^6^A was present in human sperm RNA, and asthenozoospermia patients had higher m^6^A contents. Thus, we propose that the abnormal m^6^A modification in sperm RNA may affect the steady mRNA expression of certain genes related to sperm motility, such as *cysteine-rich secretory protein 2* (*CRISP2*)^33^,^34^ and *human β-defensin 1* (*DEFB1*)^35^. However, determining which genes are responsible will require further exploration.

The discovery and characterization of m^6^A demethylases and methyltransferases and an m^6^A-selective-binding protein reveals the functional and reversible regulatory mechanism of m^6^A modification. Theoretically, the altered mRNA expression levels of demethylases, methyltransferases and m^6^A-selective-binding protein encoding genes may lead to abnormal m^6^A modification and further impact on the fundamental biological process and development of disease^36^. Our results demonstrated that *METTL3* played a major role in altering the contents of m^6^A in asthenozoospermia patients and influenced fertility. METTL3 (MT-A70) was confirmed as one component of the *N*^6^-adenosine methyltransferase complex. Bokar and colleagues^25^ isolated the methyltransferase complex from HeLa cell nuclear extracts and named the 70 kDa protein, which exhibited methyltransferase activity, MT-A70 or METTL3. Together with METTL14, another methyltransferase, METTL3 forms a stable heterodimer to mediate mammalian nuclear m^6^A methylation^16^. Interestingly, WTAP itself has no methyltransferase activity, but it can affect cellular m^6^A by interacting with the METTL3-METTL14 complex^16^. Recent studies have shown that *METTL3* participates in the regulation of a variety of life processes by catalyzing the formation of m^6^A, including stem cell self-renewal and differentiation^37^, controlling the circadian clock^38^ and marking primary microRNAs for recognition and processing^39^.

It is worth noting that *ALKBH5* deficiency can result in the elevation of m^6^A in male mice and further impair fertility^19^, whereas our data revealed that METTL3 but not ALKBH5 played a key role in human male infertility, especially in asthenozoospermia. The possible reason for this difference between humans and mice might be the diversity of the gene expression model.

Indeed, the isolation of sperm RNA is a challenging work owing to the intrinsic heterogeneous cells exist in the ejaculate and the low quantity of RNA present in Spermatozoon. Although we cannot absolutely ensure that no RNA isolated from other cellular structures in sperm purified by a typical Percoll gradient centrifugation, the potential influences are likely limited.

**Figure 3:**
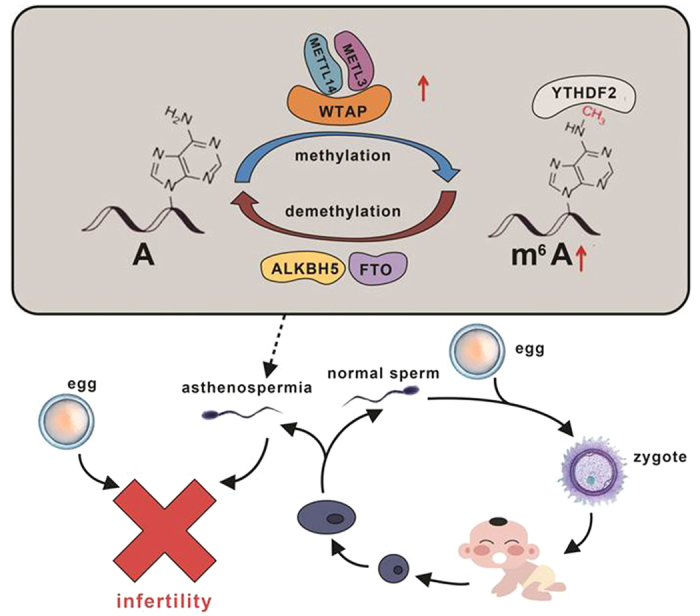
A process of the dynamic m^6^A modification in asthenozoospermia. Increased mRNA expression of *METTL3* and *METTL14* contributes to the elevation of m^6^A content which affects sperm motility and further results in male infertility.

In summary, we first reported that methyltransferases (METTL3 and METTL14), particularly METTL3, play key roles in the dynamic modification of m^6^A in asthenozoospermia patients and that increased m^6^A can impair sperm motility. According to our results, we deduce a process of dynamic modification of m^6^A in asthenozoospermia patients (Fig. 3). The underlying mechanisms of m^6^A modification in asthenozoospermia still need further investigation.

## Materials and Methods

### Chemicals and reagents

Adenosine (rA), uridine (rU), cytidine (rC), guanosine (rG), 2′-deoxyadenosine (dA), thymidine (T), 2′-deoxycytidine (dC), 2′-deoxyguanosine (dG) were purchased from Sigma-Aldrich (Beijing, China). *N*^6^-methyladenosine (m^6^A) was from Hanhong Chemical Co., Ltd. (Shanghai, China). S1 nuclease and alkaline phosphatase (CIAP) were purchased from Takara Biotechnology Co., Ltd. (Dalian, China). Phosphodiesterase I was from Sigma-Aldrich (St. Louis, MO, USA). Chloroform and formic acid were purchased from Sinopharm Chemical Reagent Co., Ltd. (Shanghai, China). Chromatographic grade methanol was purchased from Merck (Darmstadt, Germany). All water was purified by a Milli-Q apparatus (Millipore, Bedford, MA). Stock solutions of the ribonucleosides and 2′-deoxynucleosides were prepared in Milli-Q water at a concentration of 5 mmol/L.

### Semen sample collection

The ethics committee of Zhongnan Hospital of Wuhan University has approved this study. The study was carried out in accordance with the approved guidelines of the ethics committee of Zhongnan Hospital of Wuhan University and informed consent was obtained from each participant at the time of semen sample collection. According to the 5th edition of the WHO laboratory manual for the examination and processing of human semen^26^, 20 asthenozoospermia patients and 32 healthy controls from the Productive Medicine Center of Zhongnan Hospital donated semen samples by masturbation after 2–7 days of abstinence. All participants were without leukocytospermia or reproduction tract infection. To eliminate the influence of age on the results, in subjects selection we ensured no significant difference in age between healthy controls and asthenozoospermia patients (32.2 ± 5.5 years VS. 32.4 ± 4.5 years, *p* = 0.901). We defined asthenozoospermia as forward progression (progressive motility and non-progressive motility) sperm <40% and healthy control as forward progression sperm ≥40% or progressive motility sperm ≥32%. Fresh semen samples were incubated at 37 °C for 30 min to allow for liquefaction before further processing.

### Sperm purification and RNA extraction

Human sperm was purified from 2–3 mL of liquefied semen samples using density gradient centrifugation^26^. Briefly, the sample was transferred to a 15 mL conical tube (RNase-Free), and then 6–10 mL (0.01 mol/L) phosphate-buffered saline (PBS) was added and the tube inverted several times to wash the sperm. After centrifugation at 300 × g for 15 min at 4 °C, the supernatant was discarded and the sediment was resuspended in 1 mL of PBS. The sample was carefully overlaid onto a two-layer (40–80%) Percoll gradient to avoid disturbing the interface. Following another centrifugation at 400 × g for 20 min, the supernatant was discarded and the sperm pellet was resuspended in 2 mL of PBS. To confirm the purity of spermatozoa preparation, the sperms were examined by microscope(Olympus, Japan) after purification, and no lymphocytes, epithelial cells or bacteria was found under 5 microscopic vision fields (400×) (Fig. S3).Then the sperm RNA was extracted from the purified sperm suspension in a biosafety cabinet using a commercial RNAprep pure Cell/Bacteria Kit (Tiangen, Beijing, China) according to the manufacturer’s instructions. RNA was quantified by spectrophotometry (ND-2000, NanoDrop Inc., USA).

### Oligonucleotides

A 15-mer m^6^A-containing oligonucleotide was synthesized according to a previously described method^40^. The 10-mer RNA (5′-AUCUAUAUGC-3′) was purchased from Takara Biotechnology Co., Ltd. (Dalian, China).

### Enzymatic digestion of RNA

RNA (200 ng) was first denatured by heating at 95 °C for 5 min and then chilling on ice for 2 min. After adding 1/10 volume of S1 nuclease buffer (30 mM CH_3_COONa, pH 4.6; 280 mM NaCl; 1 mM ZnSO_4_) and 150 units of S1 nuclease, the mixture (20 μL) was incubated at 37 °C for 4 h. The solution was subsequently added to 1/10 volume of alkaline phosphatase buffer (50 mM Tris-HCl, 10 mM MgCl_2_, pH 9.0), 0.002 units of venom phosphodiesterase I and 15 units of alkaline phosphatase. Then, the incubation was continued at 37 °C for an additional 2 h followed by extraction with an equal volume of chloroform twice. The resulting aqueous layer was collected and lyophilized to dryness and reconstituted in 100 μL of water. Next, 30 μL of the obtained samples was subjected to liquid chromatography-electrospray ionization-tandem mass spectrometry (LC-ESI-MS/MS) analysis.

### Measurement of m^6^A content by LC-ESI-MS/MS

Analysis of nucleosides was performed on the LC-ESI-MS/MS system consisting of an AB 3200 QTRAP mass spectrometer (Applied Biosystems, Foster City, CA, USA) with an electrospray ionization source (TurboIonSpray) and a Shimadzu LC-20AD HPLC (Tokyo, Japan) with two LC-20AD pumps, a SIL-20A autosampler, a CTO-20AC thermostatic column compartment, and a DGU-20A3 degasser. Data acquisition and processing were performed using AB SCIEX Analyst 1.5 Software (Applied Biosystems, Foster City, CA). The HPLC separation was performed on a Hisep C18-T column (150 mm × 2.1 mm i.d., 5 μm, Weltech Co., Ltd., Wuhan, China) with a flow rate of 0.2 mL/min at 35 °C. Formic acid in water (0.1%, v/v, solvent A) and formic acid in methanol (0.1%, v/v, solvent B) were employed as the mobile phase. A gradient of 5 min 5% B, 10 min 5–30% B, 5 min 30–50% B, 3 min 50% B-5% B and 17 min 5% B was used.

The mass spectrometry detection was performed under positive electrospray ionization mode. The target nucleosides were monitored by multiple reaction monitoring (MRM) mode using the mass transitions (precursor ions → product ions) of m^6^A (282.2 →150.1), rA (268.4 → 136.2), rU (245.4 → 113.1), rC (244.4 → 112.2), rG (284.5 → 152.2), dA (252.4 → 136.2), T (243.3 → 127.2), dC (228.4 → 112.2), dG (268.4 → 152.4). The MRM parameters of all nucleosides were optimized to achieve maximal detection sensitivity.

### Quantification of mRNA expression by real-time PCR

For mRNA quantifications of *FTO, ALKBH5, METTL3, METTL14, WTAP* and *YTHDF2*, cDNA was synthesized by DNase treatment and reverse transcription (TOYOBO, Osaka, Japan) and real-time PCR was performed on a CFX96 Touch TM Real-Time PCR Detection System (BioRad) with iTaq TM Universal Supermixes (BioRad) and intron-spanning primers, which are listed in Table S5. The mRNA levels of the target genes were normalized to that of the reference gene *GAPDH*, and the results are expressed as the means ± SEM.

### Statistical analyses

All statistical analyses were performed using SPSS 19.0 software (SPSS Inc., Chicago, USA). A normality test was used to explore the data distribution, *p* > 0.05 was consider to be normal distributed. Two-tailed Student’s t test and Mann-Whitney U test were used to compare the differences in normal and non-normal distributed data between the two groups, respectively. The correlations of m^6^A contents with gene expression and semen parameters were assessed by Pearson correlation coefficient for normal distributed data and Spearman correlation coefficient for non-normal distributed data. Logistic regression analysis was used to evaluate whether m^6^A contents associated with asthenozoospermia risk. *p* < 0.05 was considered to be statistically significant.

## Additional Information

**How to cite this article**: Yang, Y. *et al.* Increased *N*^*6*^-methyladenosine in Human Sperm RNA as a Risk Factor for Asthenozoospermia. *Sci. Rep.* **6**, 24345; doi: 10.1038/srep24345 (2016).

## Supplementary Material

Supplementary Information

## Figures and Tables

**Table 1:** Clinical parameters of semen in controls and patients.

	Patients (n = 20)	Controls (n = 32)	*p* values
pH value	7.1 ± 0.2	7.1 ± 0.1	0.906
Concentration (10^6^/mL)	39.2 ± 23.9	96.6 ± 74.0	0.000
Progressive motility (PR, %)	8.6 ± 6.0	24.7 ± 10.2	0.000
Non-progressive motility (NP, %)	19.8 ± 7.5	44.5 ± 14.2	0.000
Immotility (IM, %)	72.4 ± 10.5	30.8 ± 13.2	0.000
Normal morphology (%)	5.1 ± 3.2	8.2 ± 4.7	0.017
Teratozoospermia index (TZI, %)	1.1 ± 0.1	1.1 ± 0.1	0.291
Sperm deformity index (SDI, %)	1.1 ± 0.1	1.0 ± 0.1	0.029

**Table 2:** Pearson correlation analysis of m^6^A content with clinical parameters of semen.

	Pearson correlation coefficient	*p* values
pH value	0.043	0.760
Concentration (10^6^/mL)	−0.238	0.089
Progressive motility (PR, %)	−0.288	0.038
Non−progressive motility (NP, %)	−0.293	0.037
Immotility (IM, %)	0.387	0.005
Normal morphology (%)	−0.039	0.792
Teratozoospermia index (TZI, %)	−0.033	0.823
Sperm deformity index (SDI, %)	0.001	0.996
