# Supplementary material for: Increased N6-methyladenosine in Human Sperm RNA as a Risk Factor for Asthenozoospermia
Source: Sci Rep. 2016 Apr 13;6:24345. doi: 10.1038/srep24345 (PMC4829835; doi:10.1038/srep24345)
Supplement: Supplementary Information [file srep24345-s1.pdf]

Increased N6-methyladenosine in Human Sperm RNA as a Risk Factor for Asthenozoospermia

Ying Yang,[1,†] Wei Huang,[2,†] Jing-Tao Huang,[1] Fan Shen,[1] Jun Xiong,[2] Er-Feng Yuan,[1] Shan-shan Qin,[2] Ming Zhang,[3] Yu-Qi Feng,[2,*] Bi-Feng Yuan,[2,*] Song-Mei Liu[1,*]

[1] Center for Gene Diagnosis, Zhongnan Hospital of Wuhan University, Donghu Road 169#, Wuhan, 430071, P.R. China

[2] Key Laboratory of Analytical Chemistry for Biology and Medicine (Ministry of Education), Department of Chemistry, Wuhan University, Wuhan, 430072, P. R. China

[3] Reproductive Medicine Center, Zhongnan Hospital of Wuhan University, Donghu Road 169#, Wuhan, 430071, P.R. China

†These authors contributed equally to this work.

*To whom correspondence should be addressed. Tel. +86-27-67812610; fax. +86-27-67812610. E-mail address: Song-Mei Liu, smliu@whu.edu.cn; Bi-Feng Yuan, bfyuan@whu.edu.cn; Yu-Qi Feng, yqfeng@whu.edu.cn.

Fig. S1. Melt curves and standard curves of real-time PCR for *METTL3* and *METTL14*. (A-B), melt curve and standard curve for *METTL3.* (C-D), melt curve and standard curve for *METTL14.*

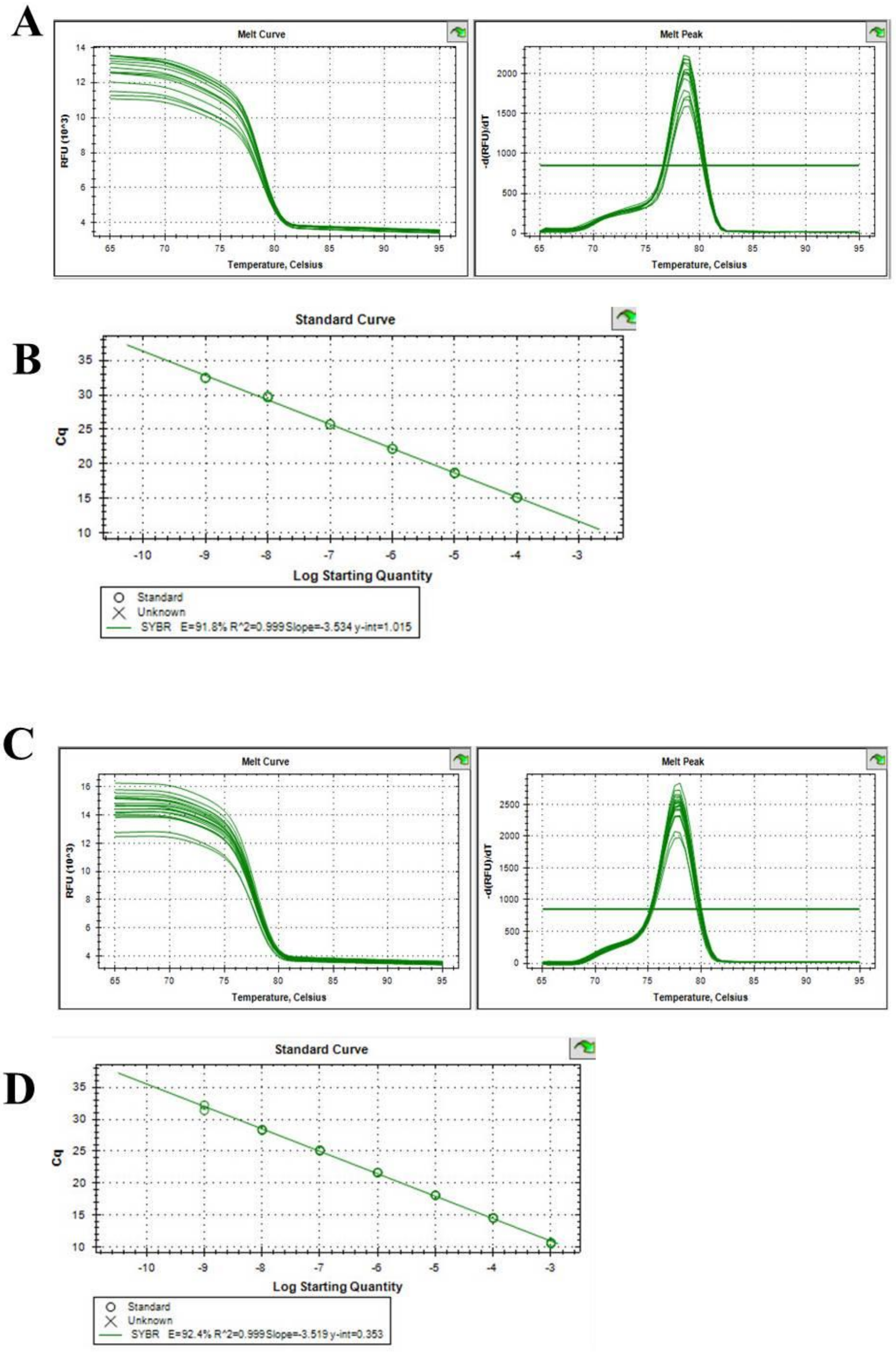

Fig. S2. Correlation of m^{6}A contents with *gene* expressions in sperm RNA. (A-E)**,** Correlation analysis between m^{6}A contents and mRNA expression in all subjects.

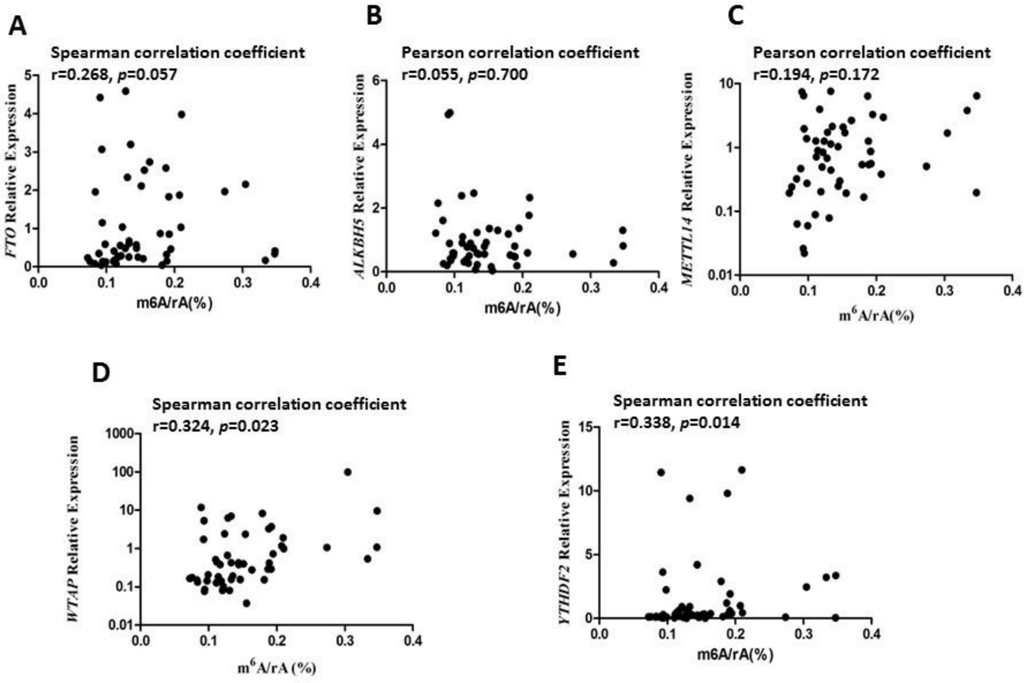

Fig. S3. Microscopic examination of the sperms before and after purification (400×).

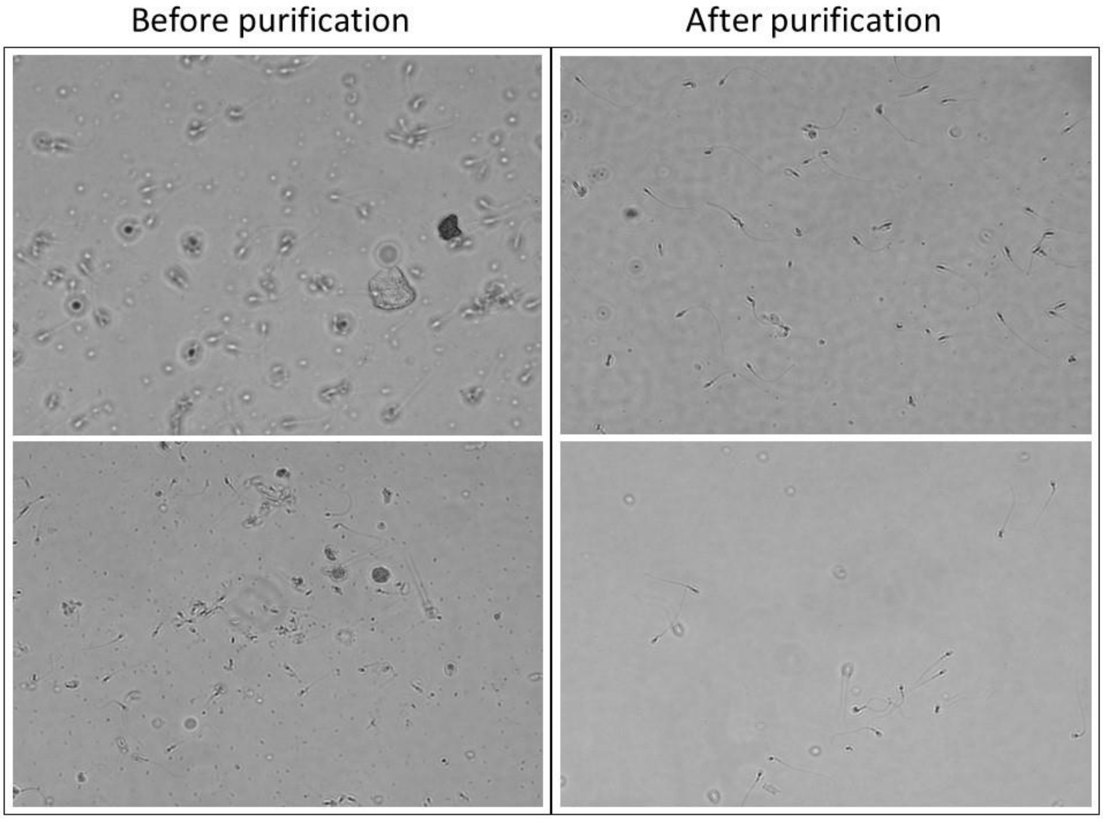

Table S1. Linearity, LOD and LOQ of m^{6}A by LC-ESI-MS/MS.

Analyte	Linear range (m^{6}A/rA,%)	Regression line		R^2	LOD (fmol)	LOQ (fmol)
		Slope	Intercept			
m^{6}A	0.02-2	0.0396 ± 0.0003	0.0012 ± 0.0001	0.9977	1.2	4.0

Table S2. Preparation of the quality control (QC) samples with m^6A-containing oligonucleotides.

Methylation level	**Molar ratio (%)**	
m^6A/A (%)	RNA standard 1	RNA Standard 2
0.050	0.16	99.84
0.100	0.30	99.70
0.300	0.91	99.09
RNA standard 1 (15-mer RNA)	5'- UAAUAC(m^6A)GAGAAAUC-3'	
RNA standard 2 (10-mer RNA)	5'-AUCUAUAUGC-3'	

Table S3. Validation of the method for the detection of m^6A with QC samples.

Nominal $[m^6A]/[A]$,%		**0.050**	**0.100**	**0.300**
Day 1 n=3	Measured mean $[m^6A]/[A]$,%	0.044	0.091	0.292
	RSD[a] (%)	5.7	4.3	1.8
	RE[b] (%)	-12.1	-9.2	-2.7
Day 2 n=3	Measured mean $[m^6A]/[A]$,%	0.055	0.093	0.321
	RSD (%)	9.7	4.6	4.4
	RE (%)	10.1	-7.4	7.1
Day 3 n=3	Measured mean $[m^6A]/[A]$,%	0.053	0.106	0.283
	RSD (%)	10.6	5.1	4.8
	RE (%)	6.4	5.6	-5.7

[a] Relative standard deviation. [b] Relative error.

Table S4. The results of m^6A contents in semen RNA

Sample Name	m6A/A(%)	SD	RSD
C1	0.15	0.007	4.69
C2	0.12	0.012	10.42
C3	0.19	0.005	2.52
C4	0.11	0.021	18.62
C5	0.08	0.005	6.72
C6	0.10	0.012	11.95
C7	0.08	0.007	8.99
C8	0.09	0.003	2.80
C9	0.14	0.001	0.85
C10	0.11	0.011	9.45
C11	0.09	0.009	9.74
C12	0.12	0.007	6.10
C13	0.13	0.014	10.56
C14	0.12	0.004	3.85
C15	0.07	0.006	8.38
C16	0.09	0.000	0.16
C17	0.11	0.006	4.98
C18	0.08	0.007	8.72
C19	0.16	0.000	0.15
C20	0.13	0.005	3.81
C21	0.19	0.029	15.28
C22	0.13	0.006	4.71
C23	0.21	0.003	1.52
C24	0.21	0.006	3.00
C25	0.16	0.012	7.85
C26	0.19	0.033	17.02
C27	0.12	0.001	0.86
C28	0.09	0.001	1.21
C29	0.21	0.005	2.21
C30	0.18	0.010	5.80
C31	0.14	0.014	9.78
C32	0.15	0.013	8.68
A1	0.35	0.010	2.95
A2	0.11	0.003	2.61
A3	0.14	0.008	5.88
A4	0.18	0.014	7.65
A5	0.13	0.000	0.19
A6	0.10	0.012	12.70
A7	0.27	0.004	1.60
A8	0.19	0.002	0.98

A9	0.19	0.011	5.58
A10	0.15	0.011	7.12
A11	0.12	0.011	8.71
A12	0.09	0.005	5.12
A13	0.33	0.029	8.61
A14	0.35	0.037	10.60
A15	0.13	0.004	3.35
A16	0.09	0.004	4.02
A17	0.10	0.012	11.89
A18	0.19	0.021	10.97
A19	0.30	0.008	2.73
A20	0.13	0.003	2.05

Table S5. Primers used for real-time PCR

Genes	Primers	
GAPDH	sense	5’-TCTATAAATTGAGCCCGCAGC-3’
	anti-sense	5’-CCAATACGACCAAATCCGTTG-3’
FTO	sense	5’-CTTCACCAAGGAGACTGCTATTTC-3’
	anti-sense	5’-CAAGGTTCCTGTTGAGCACTCTG-3’
ALKBH5	sense	5’-TCCAGTTCAAGCCTATTCG-3’
	anti-sense	5’-CATCTAATCTTGTCTTCCTGAG-3’
METTL14	sense	5’-GAGTGTGTTTACGAAAATGGGGT-3’
	anti-sense	5’-CCGTCTGTGCTACGCTTCA-3’
METTL3	sense	5’-AGATGGGGTAGAAAGCCTCCT-3’
	anti-sense	5’-TGGTCAGCATAGGTTACAAGAGT-3’
WTAP	sense	5’-TTGTAATGCGACTAGCAACCAA-3’
	anti-sense	5’-GCTGGGTCTACCATTGTTGATCT-3’
YTHDF2	sense	5’-CCTTAGGTGGAGCCATGATTG-3’
	anti-sense	5’-TCTGTGCTACCCAACTTCAGT-3’
